# Autoimmunity to selenoprotein P predicts breast cancer recurrence

**DOI:** 10.1016/j.redox.2022.102346

**Published:** 2022-05-25

**Authors:** Kamil Demircan, Qian Sun, Ylva Bengtsson, Petra Seemann, Johan Vallon-Christersson, Martin Malmberg, Lao H. Saal, Lisa Rydén, Waldemar B. Minich, Åke Borg, Jonas Manjer, Lutz Schomburg

**Affiliations:** a Institute for Experimental Endocrinology, Charité-Universitätsmedizin Berlin, Corporate Member of Freie Universität Berlin, Humboldt-Universität zu Berlin, And Berlin Institute of Health, Berlin, Germany; b Berlin Institute of Health (BIH), Biomedical Innovation Academy (BIA), Berlin, Germany; c Department of Surgery, Skåne University Hospital Malmö, Lund University, Malmö, Sweden; d selenOmed GmbH, Berlin, Germany; e Division of Oncology, Department of Clinical Sciences Lund, Lund University, Lund, Sweden; f Department of Oncology, Skåne University Hospital, Lund, Sweden

**Keywords:** SELENOP, Glutathione peroxidase, Selenium, Prognosis, Cohort study

## Abstract

**Background:**

Low concentrations of serum selenium (Se) and its main transporter selenoprotein P (SELENOP) are associated with a poor prognosis following breast cancer diagnosis. Recently, natural autoantibodies (aAb) with antagonistic properties to SELENOP uptake have been identified in healthy subjects, and in patients with thyroid disease. Given the potential transport disrupting properties, we hypothesized that breast cancer patients with SELENOP-aAb may have a poor prognosis.

**Methods:**

SELENOP-aAb along with serum Se, SELENOP and GPX3 activity were determined in serum samples of 1988 patients with a new diagnosis of breast cancer enrolled in the multicentre SCAN-B study. Patients were followed for ∼9 years and multivariate Cox regression models were applied to assess hazard ratios.

**Results:**

Applying a cut-off based on outlier detection, we identified 7.65% of patients with SELENOP-aAb. Autoantibody titres correlated positively to total Se and SELENOP concentrations, but not to GPX3 activity, supporting a negative role of SELENOP-aAb on Se transport. SELENOP-aAb were associated with age, but independent of tumor characteristics. After fully adjusting for potential confounders, SELENOP-aAb were associated with higher recurrence, HR(95%CI) = 1.87(1.17–2.99), particularly in patients with low Se concentrations, HR(95%CI) = 2.16(1.20–3.88). Associations of SELENOP-aAb with recurrence and mortality were linear and dose-dependent, with fully adjusted HR(95%CI) per log increase of 1.25(1.01–1.55) and 1.31(1.13–1.51), respectively.

**Conclusion:**

Our results indicate a prognostic and pathophysiological relevance of SELENOP-aAb in breast cancer, with potential relevance for other malignancies. Assessment of SELENOP-aAb at time of diagnosis identifies patients with a distinctly elevated risk for a poor prognosis, independent of established prognostic factors, who may respond favourably to Se supplementation.

## Highlights

• SELENOP-aAb correlated with higher serum Se and SELENOP, but not GPX3, indicating a transport/uptake disrupting role.
• SELENOP-aAb associated with poor prognosis following breast cancer diagnosis, independent of established prognostic factors.
• SELENOP-aAb correlated with mortality and recurrence in a dose-dependent manner.
• The association with poor prognosis was distinct in Se/SELENOP deficiency.

## Graphical abstract

**Figure:**
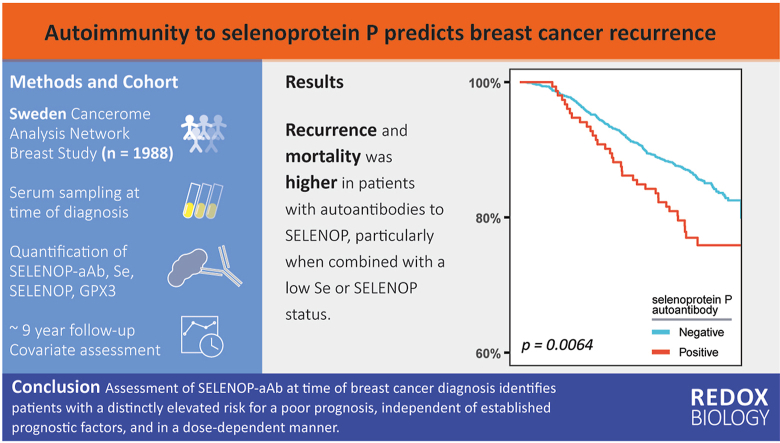

## Abbreviations

BI: binding index
CI: confidence interval
CV: coefficient of variation
ER: oestrogen receptor
GPX3: glutathione peroxidase
HER2: human epidermal growth factor receptor
HR: hazard ratio
IQR: interquartile range
Ki67: Kiel-antigen nr. 67
NHG: Nottingham Histological Grade
NKBC: Swedish National Quality Registry for Breast Cancer
OS: overall survival
PGR: progesterone receptor
PMM: predictive mean matching
RCS: restricted cubic spline
RFI: recurrence free interval
RLU: relative light units
SCAN-B: Swedish Cancerome Analysis Network – Breast
SEAP: secreted alkaline phosphatase
SELENOP: selenoprotein P
SELENOP-aAb: selenoprotein P autoantibodies
Se: Selenium
TXRF: total x-ray fluorescence

## 1 Introduction

Breast cancer accounts for one quarter of all cancers, and one sixth of all cancer deaths in women [1]. Given the high incidence, most effort for reducing mortality over recent years has been put on early detection with screening programs [2,3]. However, the established prognostic factors including mainly tumor characteristics (histological grade, receptor expression) and tumor stage remained widely unchanged. Discovery of additional factors for the early identification of patients at high risk for breast cancer recurrence and subsequent intensified adjuvant therapy may improve prognosis.

The trace element selenium (Se) is essential for life, owing to its effects executed as active constituent of selenoproteins [4,5]. Mainly due to the function of several of the selenoproteins controlling redox status, antioxidative reactions and protective pathways, a beneficial role of Se for maintaining health and avoiding disease has been discussed since more than 40 years [6,7]. While no consistent results were obtained for cancer incidence [[8], [9], [10]], several independent studies reported dose-dependent associations of low Se status with poor prognosis. An inverse association of Se status with cancer-prognosis is described for multiple cancer sites, including laryngeal [11], colorectal [12,13], lung [13,14], prostate [13], skin [15], and breast [[16], [17], [18], [19], [20]], and it was also observed in large-scale studies assessing all-cancer mortality including NHNAES III [13].

Most of the studies that analysed prognosis of patients with breast cancer by Se used blood sampling to determine Se concentrations in serum or plasma. In our recent study, the association with prognosis was assessed using three different serum biomarkers, namely total Se, the Se transport protein selenoprotein P (SELENOP), and the enzymatic activity of extracellular Se-dependent glutathione peroxidase (GPX3). All three biomarkers were inversely associated with prognosis. Besides these interrelated biomarkers of Se status, natural autoantibodies to SELENOP (SELENOP-aAb) have recently been reported in healthy subjects and thyroid patients, obviously capable of interfering with regular Se transport by SELENOP [21].

The aim of the study was to assess the prognostic value of SELENOP-aAb in a large multicentre population-based cohort of newly diagnosed breast cancer patients.

## 2 Methods

### 2.1 Study population

Since August 30^th^ 2010, the multicentric prospective Sweden Cancerome Analysis Network – Breast (SCAN-B) study (ClinicalTrials.gov ID NCT02306096) enrols patients with a new diagnosis of primary invasive breast cancer systematically, with the aim of identifying novel genomic and serum prognostic factors [16,22,23]. With multiple participating hospitals in Sweden in Malmö, Lund, Helsingborg, Kristianstad, Växjö, Halmstad, Uppsala, Karlskrona, Varberg, and Ljungby, SCAN-B included almost 85% of all cases in the catchment region since its initiation [22]. Patients with a pre-surgical diagnosis or suspicion of primary invasive breast cancer were eligible. Among this group, patients with a previous history of contralateral breast cancer, without planned treatment, without planned treatment in any of the participating hospitals, with an unclear treatment status or with a generalized disease state at time of diagnosis, i.e. with distant metastases, were excluded. A total of 5417 patients meeting the eligibility and exclusion criteria were registered between September 1^st^ 2010 and March 31^st^ 2015. For the purpose of our study, we aimed to include 2000 patients. Hence, the first 2903 consecutive cases were selected. After excluding 915 cases, mainly due to missing serum, samples of 1988 female patients were finally included in the current analyses (Supplementary Fig. 1).

### 2.2 Follow up and endpoint retrieval

For all patients, follow-up started at time of diagnosis and serum sampling, before initiation of surgical treatment. Patients were followed until death, recurrent event (local, regional, distant), or end of follow up. In order to maintain and protect patient confidentiality, the SCAN-B steering committee provided only the number of follow-up days to the authors, instead of exact date of follow-up start and date of event of interest. Thus, end of follow-up time is a date between April 1^st^ 2019 and June 30^th^ 2019. Retrieval of endpoint data in the case of recurrence and all-cause mortality was conducted by linkage with the Swedish National Quality Registry for Breast Cancer (NKBC). NKBC retrieves mortality data from the Swedish Population Registry, and recurrence data from reports of treating centres.

### 2.3 Clinical data and tumor characteristics

Clinical data and tumor characteristics collected by the surgical and pathological department of each participating centre were obtained from the NKBC. Patient-related data comprised age, sex, and menopausal state if applicable. Tumor characteristics as assessed for the purpose of this study were size, histopathological type, Nottingham Histological Grade, Ki67 expression, oestrogen receptor overexpression, progesterone receptor overexpression, HER2 receptor overexpression, and lymph node involvement.

### 2.4 Modality of diagnosis and treatment

Data on diagnosis modality, surgical procedure with regard to the breast and with respect to the axilla, adjuvant endocrine therapy, chemotherapy, immunotherapy, and/or radiotherapy were reported to and retrieved from NKBC.

### 2.5 Quantification of selenium status biomarkers

Serum sampling was conducted within the SCAN-B infrastructure. In brief, blood was drawn at time point of breast cancer diagnosis, before initiation of treatment, and 200 μL aliquots of serum were prepared and kept at −80 °C at the Department of Clinical Chemistry, Skåne University Hospital. The laboratory analyses took place in an off-site laboratory in Berlin, Charité University, Germany, while clinical data was entirely blinded to the receiver of the samples as well as to scientists and technicians conducting laboratory analyses. Linkage of the results to clinical phenotype, i.e. unblinding took place after all measurements were completed, and no additional quantification was conducted after unblinding.

Three complementary Se status biomarkers in the serum samples, i.e., total serum Se and SELENOP concentrations along with GPX3 enzyme activity, have been assessed and were described earlier [16]. Total reflection X-ray fluorescence (TXRF) was used for total serum Se [24], a validated sandwich ELISA (selenOtest™-ELISA,selenOmed GmbH, Berlin, Germany) for serum SELENOP concentrations [25,26], and an NADPH-coupled enzymatic test for serum GPX3 activity [27,28]. Inter- and intraassay coefficients of variation were below 15% at all times, as reported earlier [16].

### 2.6 Assessment of SELENOP autoantibodies

Natural SELENOP-aAb in the serum samples were detected and assessed as described recently [21]. Briefly, serum samples were incubated with a fusion protein consisting of a secreted alkaline phosphatase (SEAP) fused in frame to recombinant SELENOP variant in which selenocysteine has been replaced by cysteine residues as reporter (SEAP-SELENOP, selenOmed GmbH). Samples were incubated overnight at 4 °C, and the immune complexes formed (SELENOP-aAb) bound to SEAP-SELENOP fusion protein) were precipitated with protein A-sepharose, washed and analysed for SEAP activity in a luminometer. Luminescence corresponding to SELENOP-aAb concentration in the original sample is recorded as relative light units (RLU), and analysed in relation to background signals. Inter- and intra-assay CV using a positive sample as standard were below 15% and 11%, respectively, during the analyses.

### 2.7 Statistical analysis

#### 2.7.1 Classification of autoimmunity to SELENOP

Patients were assigned as SELENOP-aAb positive or negative based on the signals obtained from serum by assessing the binding of immunoglobulins to recombinant SELENOP as described above. Final classification as positive or negative was carried out applying a mathematical outlier criterion. Based on the assumption of SELENOP-aAb being prevalent in less than 50% of samples, the arithmetic mean of the low 50% of signals per measurement plate was calculated, defined as background and assigned as a binding index (BI) of BI = 1. All values equal or above 3-fold of this signal, i.e. BI ≥ 3 were considered positive. Distribution of the resulting BI of single 96-well plates and the full set of results was assessed by dot-plots and density plots.

#### 2.7.2 Autoimmunity to SELENOP in relation to baseline patient and tumor characteristics

Baseline patient and tumor characteristics were described as mean (standard deviations) in case of normal, or as median (interquartile range) in case of non-normal distribution. Distribution was evaluated based on the Shapiro-Wilk test and visual inspection of histogram plots. Patient characteristics were compared in relation to SELENOP-aAb. Wilcoxon rank sum test was used to test differences in continuous variables, Fisher's exact test was used to test differences between categorical variables in a 2 × 2 contingency format, and Pearson's Chi-squared test was used to test differences in categorical variables with more categories.

#### 2.7.3 Correlation of SELENOP-aAb to Se status biomarkers

Correlation between SELENOP-aAb and total Se or selenoproteins was tested with Spearman's rank correlation, and a visual trend was investigated by linear regression plots with 95% confidence intervals (CI). A potential dose-dependent relationship was assessed by applying different cut-offs for the signal strength, i.e., BI ≥ 3, BI ≥ 5 and BI ≥ 10, respectively.

#### 2.7.4 SELENOP-aAb in relation to mortality and recurrence

Prognosis was assessed based on overall survival (OS) and recurrence free interval (RFI). Starting time-point of the follow-up for both endpoints was the time at diagnosis, before surgery. Mortality of any cause was the event for OS. Breast cancer recurrence (local, regional or distant) was the event for RFI, while death was censored. Survival probability was visualized with Kaplan-Meier plots, and the log-rank test was used to detect differences between SELENOP-aAb positive and negative patients. Cox regression models were conducted to calculate HR and 95% confidence interval (CI). Proportional hazards assumption was checked by visual inspection of Kaplan-Meier plots and by computing Schoenfeld residuals (Supplementary Fig. 2), without observing any violations. Three models were applied. First model was univariate, the second model was adjusted for age at diagnosis, and the third model was additionally adjusted for various potential confounders of mortality and recurrence, including Nottingham Histologic Grade (NHG), histological type of the tumor, expression of HER2 receptor, progesterone receptor, or oestrogen receptor, tumor size, modality of diagnosis of breast cancer, and menopausal state of the patient. As Ki67 evaluation was not part of clinical routine, Ki67 variable has high number of missing values and was not included in the model. In all analyses, the negative patient group was set as reference. Dichotomizing a continuous variable makes it easy to interpret and apply the parameter in clinical decision making. However, statistical power is sacrificed, and a dose-dependent relationship – which is a factor suggesting a causal relationship – cannot be investigated [29,30]. Therefore, SELENOP-aAb concentrations were also modelled as a continuous variable in relation to OS and RFI using linear Cox regression. As the values were right skewed, the variable was log-transformed applying natural logarithm to approximate a normal distribution. Shape of association was assessed with restricted cubic spline regression (RCS) modelling. Three knots at the 10^th^, 50^th^ and 90^th^ percentile were fitted to the RCS models. RCS models were compared to linear models by applying likelihood-ratio test and p-value for non-linearity was evaluated.

#### 2.7.5 Evaluation and handling of missing data

As described previously, the fraction of the missing data included in the models constituted less than 1% of all data [16]. When applying fully adjusted models, those were imputed by multiple chained imputation, applying ten imputations iterated 10 times each. All variables included in the fully adjusted model as well as total Se, SELENOP, GPX3, both outcome measures and time from diagnosis to endpoint were entered into the prediction matrix. Fully conditional specification was applied with proportional odds model for ordinal variables, predictive mean matching (PMM) for continuous variables, logistic regression for binary categorical covariates, and polytomous logistic regression for nominal data. Robustness of the imputation model was solid, as assessed by checking convergence, as well as comparing regression results to complete case analysis, as shown before [16].

#### 2.7.6 Subgroup and sensitivity analyses

The association of positivity and SELENOP-aAb titres with mortality and recurrence was tested in patients with low and high SELENOP concentrations separately. For that purpose, the cohort was divided into two groups based on the median value of SELENOP, which equals 4.08 mg/L. All analyses were also conducted in low and high total serum Se concentration groups, where the median Se corresponds to 70.4 μg/L. Association of SELENOP-aAb with mortality and recurrence was repeated in the fully adjusted models, by adding serum Se status biomarkers one by one. In order to rule out potential reverse causality, the main analyses were repeated excluding patients with an event or censoring within the first 12 months of follow up. In a further sensitivity analysis, the fully adjusted main analyses for all surgical and adjuvant treatment options was adjusted one-by-one to detect potential adjustment effects by treatment modality.

All statistical analyses were two-sided and were conducted with the R language (version 4.1.2.) on the RStudio environment. Packages used for main analyses are provided in the supplement section.

## 3 Results

### 3.1 Study design and prevalence of autoantibodies to SELENOP

**Fig. 1:**
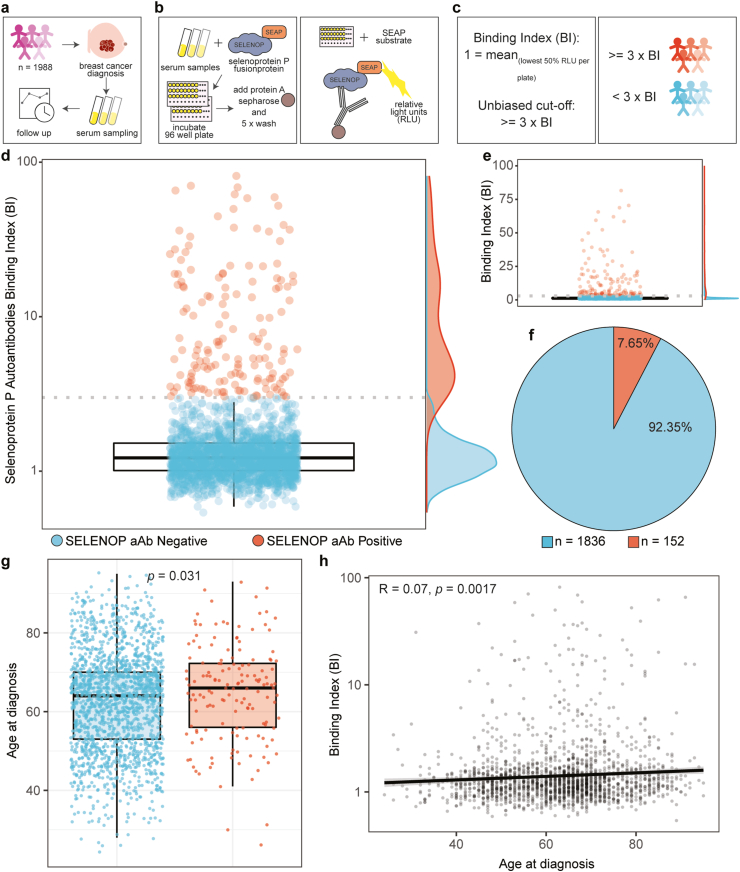
**Study design and prevalence of autoimmunity to SELENOP. a** 1988 patients with an incident diagnosis of primary invasive breast cancer were included in this study. Serum sampling was conducted at time of diagnosis, and follow up encompassed approximately 9 years **b** Samples were analysed for SELENOP-aAb in 96 well plates by immunoprecipitation of complexes formed in serum with protein A-sepharose, and detection of luminescence as light units (RLU) from precipitated SELENOP-SEAP-aAb complexes. **c** An outlier criterion for cut-off definition of autoimmunity was applied, and values exceeding 3-fold of binding index (BI ≥ 3, dotted line) were considered positive. **d** Binding indices of SELENOP-aAb are displayed on a logarithmized y-axis, and plotted as density on the right y-axis. Patients above the cut-off are marked red **e** SELENOP-aAb displayed a right skew, as emphasized by the marginal density plot. BI was displayed on non-logarithmized y-axis. **f** Applying the unbiased cut-off (BI ≥ 3), a total of 7.65% of patients were identified as SELENOP-aAb positive. **g** Age at diagnosis was compared to aAb-positivity, applying the Wilcoxon-Rank-sum test. **h** Correlation of the continuous SELENOP-aAb titre and age at diagnosis was assessed, using Spearman's rank correlation test. Blue points indicate SELENOP-aAb negative patients, and red points indicate SELENOP-aAb positive patients. (For interpretation of the references to color in this figure legend, the reader is referred to the Web version of this article.)

Final analysis comprised 1988 patients with an incident diagnosis of primary invasive breast cancer. Serum sampling was conducted for each patient at time of breast cancer diagnosis, before surgical intervention (Fig. 1a). The follow-up time corresponded to a median (IQR) of 6.94 (6.28–7.63) years for OS comprising 13,290 person years, and 6.87 (6.25–7.61) years for RFI comprising 13,023 person years. In total, 307 deaths and 167 recurrent events occurred during the follow-up.

The quantification of SELENOP-aAb in serum was conducted via protein A-mediated precipitation of a recombinant SEAP- SELENOP fusion protein (Fig. 1b). Serum samples were assessed for their signal in relation to background, with signals exceeding three times background signal (BI ≥ 3) classified as outliers and SELENOP-aAb positive samples (Fig. 1c). The signals obtained showed a skewed distribution (Fig. 1d). This result is highlighted by the dot-plot and marginal density plot analysis presented (Fig. 1e). The unbiased cut-off (depicted by the dotted grey line in Fig. 1d and **e**) was in agreement with an alternative outlier criterion (3^rd^ quartile + 1.5 * interquartile range), which is depicted by the upper whisker of the black boxplot (Fig. 1d). According to this analysis, the prevalence of SELENOP-aAb in the full set of samples was 7.65% (152/1988), including a fraction of 3.05% (61/1996) with particularly high titres of BI ≥ 10 (Fig. 1f). Age of patients was higher in the SELENOP-aAb positive group (Fig. 1g), with a weak correlation of the BI to age (Fig. 1h).

### 3.2 SELENOP autoantibodies are associated with higher serum SELENOP but not higher GPX3 expression

**Fig. 2:**
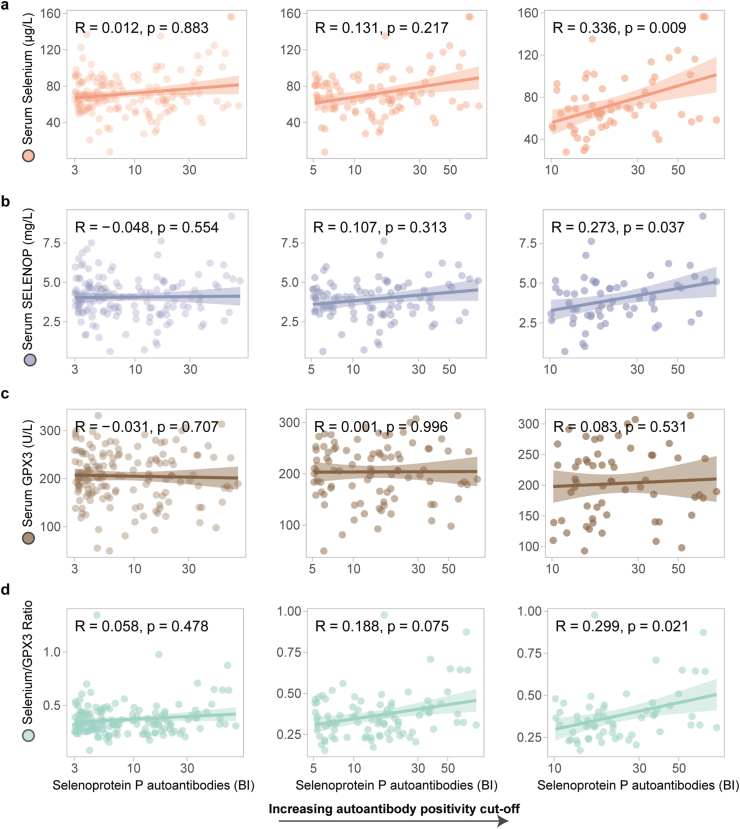
**Correlation of SELENOP-aAb with total serum selenium and selenoproteins.** Linear regression (line) with 95% confidence intervals (shadow) was used to visualize the relationship. **a** Correlation of autoantibody titres to total serum selenium was assessed, with increasing cut-offs for autoantibody titres from left to right. Slope of the linear regression line has shown an increasing trend with increasing antibody titres. Above BI = 10, SELENOP-aAb were significantly correlated with total serum selenium, R = 0.336, p = 0.009. **b** A similar trend was seen with regard to serum SELENOP levels, which also was statistically significant above BI = 10, R = 0.273, p = 0.037. **c** No association was seen for serum GPX3, although it is tightly correlated to serum selenium and serum SELENOP concentrations in this study cohort. **d** SELENOP-aAb were significantly associated with selenium/GPX3 ratio above BI = 10, R = 0.299, p = 0.021. Spearman's rank correlation was used to assess correlation.

A potential dose-dependent association of SELENOP-aAb with total Se, SELENOP and GPX3 was tested next (Fig. 2). SELENOP-aAb were dose-dependently correlated to serum SELENOP and total serum Se concentrations, with an increasing gradient over increasing autoantibody titres. Above a cut-off of BI = 10, this association was significant, R = 0.336, p = 0.007 (total Se) (Fig. 2a), and R = 0.273, p = 0.037 (SELENOP) (Fig. 2b). No association was observed for GPX3 activity in relation to SELENOP-aAb (Fig. 2c), supporting a role of SELENOP-aAb in disruption of Se transport. This notion was supported by a stringent association of SELENOP-aAb with the Se/GPX3 ratio (Fig. 2d).

### 3.3 Tumor characteristics do not differ according to SELENOP autoimmunity

Patient and tumor characteristics were analysed with respect to SELENOP-aAb (Table 1). On average (median(IQR)), SELENOP-aAb positive patients were older at time of diagnosis than negative patients, 66 (56–72) vs. 64 (53–70) years. Classical tumor characteristics including Nottingham Histologic Grade, expression status of common receptors (ER, PGR, HER2), tumor size, or lymph node involvement did not differ between SELENOP-aAb positive and negative patients. Similarly, the mode of diagnosis, the surgical method conducted with regard to the breast or lymph nodes as well as the applied adjuvant therapy (chemo-, radio-, immune-, or endocrine therapy) did not differ according to SELENOP-aAb status (Supplementary Table 1).

**Table 1:** Patient and tumor characteristics in relation to SELENOP-aAb positivity.

Characteristic	SELENOP-aAb negative N = 1836	SELENOP-aAb positive N = 152	p-value^a^
***Age (years)***	*64 (53, 70)*	*66 (56, 72)*	0.031
**Menopausal Status**			0.4
Pre-menopausal	343 (19%)	22 (15%)	
Post-menopausal	1401 (77%)	123 (81%)	
Uncertain	77 (4.2%)	6 (4.0%)	
**Laterality**			0.063
Left	943 (51%)	90 (59%)	
Right	893 (49%)	62 (41%)	
***Size (mm)***	16 (11, 23)	15 (10, 22)	0.2
**Lymph Nodes**			0.3
≥4	164 (9.3%)	10 (6.9%)	
1-3	430 (24%)	29 (20%)	
No Involvement	1134 (64%)	101 (70%)	
Submicrometastasis	37 (2.1%)	5 (3.4%)	
(Missing)	71	7	
**NHG**			0.4
I	348 (19%)	35 (24%)	
II	846 (47%)	68 (47%)	
III	591 (33%)	43 (29%)	
(Missing)	51	6	
**Ki67 Expression**			0.2
Low	203 (46%)	22 (58%)	
High	236 (54%)	16 (42%)	
(Missing)	1397	114	
**Histological Type**			0.087
Ductal	1467 (80%)	122 (80%)	
Lobular	245 (13%)	15 (9.9%)	
Other	96 (5.2%)	9 (5.9%)	
Ductal + Lobular/Other	26 (1.4%)	6 (3.9%)	
**HER2 Expression**			0.7
Negative	1586 (87%)	128 (86%)	
Positive	227 (13%)	20 (14%)	
**ER Expression**			0.2
Negative	254 (14%)	27 (18%)	
Positive	1578 (86%)	124 (82%)	
**PGR Expression**			>0.9
Negative	514 (28%)	43 (28%)	
Positive	1318 (72%)	108 (72%)	

*Median (IQR)*; n (%).

Missing not shown if <2%.

NHG = Nottingham Histological Grade, Lymph Nodes = Number of lymph nodes involved, HER2 = Human epidermal growth factor receptor 2, ER = Oestrogen receptor, PGR = Progesterone receptor.

a Wilcoxon rank sum test; Pearson's Chi-squared test; Fisher's exact test.

### 3.4 SELENOP autoantibodies are associated with poor breast cancer prognosis

**Fig. 3:**
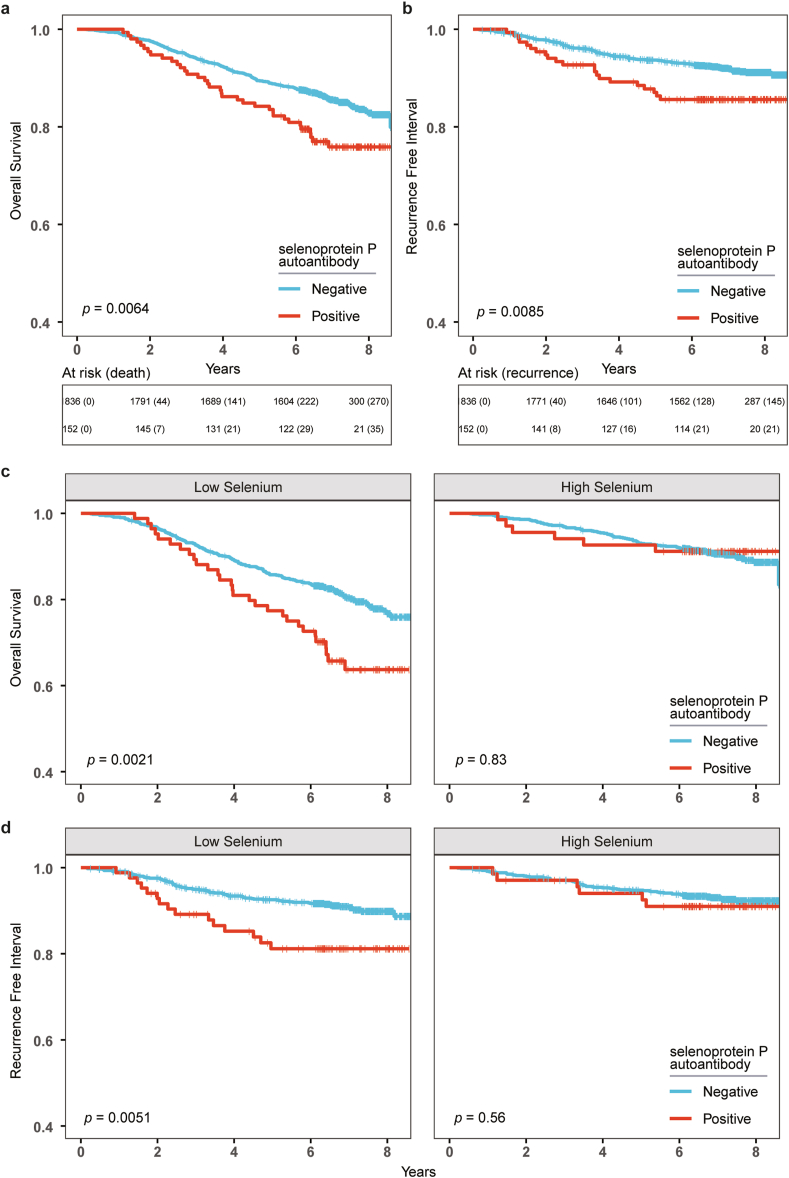
**Kaplan Meier plots for overall survival and recurrence free interval. a** Overall survival according to autoantibody positivity was assessed with Kaplan Meier plots and log-rank test. Overall survival differed significantly between the two groups. **b** Recurrence free interval was also lower in SELENOP-aAb positive patients. **c** Overall survival probability stratified by Se status, cut-off was set at median of the cohort, corresponding to 70.4 μg/L Se. **d** Recurrence free interval stratified by Se status.

Survival probability was compared between patients positive and negative for SELENOP-aAb using Kaplan-Meier plots (Fig. 3). OS probability was significantly lower in SELENOP-aAb positive as compared to SELENOP-aAb negative patients, log-rank p = 0.0064 (Fig. 3a). RFI was also lower in patients positive for SELENOP-aAb, log-rank p = 0.0085 (Fig. 3b). Cox regression analyses were carried out for OS and RFI in relation to SELENOP-aAb (Table 2). Three models were fit to assess the hazard ratio, namely univariate, age adjusted and fully adjusted. Patients negative for SELENOP-aAb were set as reference. In univariate models, HR for mortality (OS) and recurrence (RFI) was significantly higher in SELENOP-aAb positive patients, HR = 1.62 (95% CI = 1.14 to 2.31) and HR = 1.83 (95%CI = 1.16 to 2.89), respectively. HR for RFI remained significantly elevated in the age adjusted and fully adjusted models, and HR for OS was borderline significant after full adjustment (Table 2).

**Table 2:** Cox regression according to positivity of autoantibodies in the whole cohort.

		At Risk	Event	Univariate^a^		Age Adjusted^b^		Fully Adjusted^c^	
Endpoint	SELENOP-aAb	N	N	HR	95% CI	HR	95% CI	HR	95% CI
**Mortality**									
	Negative	1836	272	—	—	—	—	—	—
	Positive	152	35	1.62	1.14, 2.31	1.45	1.02, 2.06	1.41	0.98, 2.02
**Recurrence**									
	Negative	1836	146	—	—	—	—	—	—
	Positive	152	21	1.83	1.16, 2.89	1.79	1.13, 2.84	1.87	1.17, 2.99

HR = Hazard Ratio, CI = Confidence Interval.

a Crude model. Complete case.

b Adjusted for age at diagnosis. Complete Case.

c Fully Adjusted Model. Missing covariates were imputed using multiple imputation by chained equations. Adjusted for age at diagnosis, menopausal Status, ER expression, PGR expression, HER2 expression, Nottingham Histologic Grade, histological type, number of lymph nodes involved, modality of diagnosis, and size of tumor [mm].

### 3.5 Association of SELENOP autoantibodies and prognosis in relation to selenium deficiency

In the low Se group, the OS was significantly lower for SELENOP-aAb positive patients as compared to SELENOP-aAb negative patients (log-rank p = 0.0021), while OS did not statistically differ in the high Se group (Fig. 3c). Similarly, RFI probability of positive patients was significantly lower than of negative patients only in the low Se group (log-rank p = 0.0051) (Fig. 3d). Next, Cox regression was implemented to adjust for confounders. The observed differences were retained in fully adjusted models, and HR for RFI in positive patients was 2.16 (95%CI = 1.20 to 3.88) and for OS 1.58 (95% CI = 1.04 to 2.40) in the low Se group (Table 3).

**Table 3:** Cox regression according to positivity of autoantibodies stratified by selenium status.

		At Risk	Event	Univariate^a^		Age Adjusted^b^		Fully Adjusted^c^	
Group (Endpoint)	SELENOP aAb	N	N	HR	95% CI	HR	95% CI	HR	95% CI
Low Selenium **Mortality**									
	Negative	908	181	—	—	—	—	—	—
	Positive	84	29	1.83	1.23, 2.72	1.65	1.11, 2.45	1.58	1.04, 2.40
High Selenium **Mortality**									
	Negative	928	91	—	—	—	—	—	—
	Positive	68	6	1.18	0.61, 2.27	0.87	0.38, 2.00	0.88	0.38, 2.06
Low Selenium **Recurrence**									
	Negative	908	81	—	—	—	—	—	—
	Positive	84	15	2.16	1.23, 3.77	2.11	1.21, 3.69	2.16	1.20, 3.88
High Selenium **Recurrence**									
	Negative	928	65	—	—	—	—	—	—
	Positive	68	6	0.83	0.29, 2.32	1.27	0.54, 2.98	1.25	0.53, 2.97

HR = Hazard Ratio, CI = Confidence Interval.

a Crude model. Complete case.

b Adjusted for age at diagnosis. Complete Case.

c Fully Adjusted Model. Missing covariates were imputed using multiple imputation by chained equations. Adjusted for age at diagnosis, menopausal Status, ER expression, PGR expression, HER2 expression, Nottingham Histologic Grade, histological type, number of lymph nodes involved, modality of diagnosis, and size of tumor [mm].

In a sensitivity analysis, we evaluated OS and RFI stratified by total SELENOP concentrations, which yielded very similar results (Supplementary Fig. 3). After full adjustment, HR for RFI (Supplementary Table 2) and OS (Supplementary Table 3) was strongly elevated in SELENOP-aAb positive as compared to SELENOP-aAb negative patients in the low SELENOP group.

### 3.6 Association of autoantibody titres with poor prognosis is dose-dependent

SELENOP-aAb concentrations were modelled as a continuous variable in relation to OS and RFI using linear Cox regression to evaluate a potential dose-dependent relationship. After full adjustment for confounders, one natural logarithmic increase of SELENOP-aAb titres was associated with an HR of 1.31 (1.13–1.51) for OS and 1.25 (1.01–1.55) for RFI, when including the whole cohort (Supplementary Table 4). All associations assessed were of linear shape, i.e. p_non-linearity_ > 0.05 (Supplementary Fig.4). The association of SELENOP-aAb and mortality or recurrence was very similar after adjusting for any of the other Se biomarkers one-by-one or all together (Supplementary Table 5).

When stratified for SELENOP concentrations, the continuous variable SELENOP-aAb was associated with OS and RFI in the low SELENOP group. HR per log increase was 1.32 (95% CI = 1.10 to 1.58) for mortality, and 1.37 (95%CI = 1.06 to 1.77) for breast cancer recurrence in the fully adjusted model (Supplementary Table 6). Conducting the linear Cox regressions analyses stratified by total Se concentrations yielded very similar results (Supplementary Table 7).

In order to investigate a potential reverse causality of the effects, fully adjusted models for SELENOP-aAb and the continuous autoantibody variable in relation to OS and RFI were repeated, excluding patients with an event/censoring within the first 12 months. Autoimmunity or log increase in continuous variable remained significantly associated with both mortality and recurrence (Supplementary Table 8).

For the purpose of assessing potential adjustment effects of surgical and adjuvant therapy to the association, fully adjusted models were augmented with each treatment method one at a time, without observing considerable changes in the HR (Supplementary Table 9).

## 4 Discussion

In this manuscript, we describe the prognostic relevance of autoimmunity to the Se transporter SELENOP in patients with a new diagnosis of primary invasive breast cancer. The association of SELENOP-aAb with poor prognosis was most distinct in Se deficient patients. Patients positive for SELENOP-aAb displayed elevated total Se and SELENOP concentrations in serum, but no elevated GPX3, indicating a disrupting effect of the autoantibodies on regular Se transport and homeostasis. The potential causality is supported by the dose-dependent relationship between SELENOP-aAb concentration and mortality or recurrence, which maintained after adjusting for potential confounders of breast cancer prognosis, and the other three biomarkers of Se status. We conclude that an assessment of SELENOP-aAb identifies patients at high risk for breast cancer recurrence, independent of the commonly assessed prognostic factors.

Beside the need for further studies with regard to risk of developing breast cancer, our results are highly congruent for survival, the main objective of our study. In line with prior observations of the inverse association of Se status biomarkers and mortality/recurrence following breast cancer, and in line with potential antagonistic properties of the SELENOP-aAb, we have observed a poor prognosis in SELENOP-aAb positive patients. Even though our study is investigating this matter for the first time, our results are backed up by several supportive backbones. Firstly, our study describes the postulated occurrence of autoimmunity to SELENOP in female patients, with an expected association to higher patient age [31]. Secondly, the autoantibodies were dose-dependently associated to higher SELENOP and Se concentrations, without a rise in GPX3 activity, which is mainly controlled by SELENOP-dependent Se supply to the kidney [32]. This is in line with our previous study in an independent cohort, and supports the hypothesis of potential antagonistic properties of the autoantibodies to Se uptake [21]. Thirdly, equal to the three Se status biomarkers, SELENOP-aAb were not related to any tumor characteristics, only to age of patients at diagnosis. These three points are coherent in themselves, and support the quality of the quantitative analysis of the main exposure, SELENOP-aAb. Further, the association with prognosis was particularly severe in patients with low serum concentrations of SELENOP, which accords with the hypothesis that SELENOP-aAb bind and inhibit uptake of SELENOP in a dose-dependent matter. Lastly, modelling the autoantibody titres as a continuous variable revealed a dose-dependent relationship of SELENOP-aAb with prognosis, similar to the observations with the other Se status biomarkers. The observed dose-dependency argues against a chance finding, and supports a potential causal relationship.

Current prognostic factors with an established clinical role mostly require invasive methods and sampling of tumor tissue, e.g., immunohistochemical, gene expression profile or epigenetic pattern analyses [[33], [34], [35]]. The assessment of SELENOP-aAb at the time of cancer diagnosis offers some promising perspectives, as the biomarker would be accessible directly from a serum sample, requiring little volume only, and would not depend on very elaborate, cost- or labour-intensive instrumentation. Still, the robustness and reproducibility of the results presented needs some independent replication in additional sufficiently-large cohort studies.

The SCAN-B study is fully integrated into the clinical routine with a high rate of coverage of all breast cancer cases in Southern Sweden, with all procedures regarding diagnosis and treatment proceeding regularly, without alterations in clinical decision making [22]. Thus, a high generalisability of the results is ensured with regard to study characteristics, coverage and design. However, although a considerable part has non-European origin, majority of patients is genetically similar and of European origin, environmental factors and nutritional patterns are similar, and the Se status of the population is accordingly marginal, similar to other European countries [[36], [37], [38]]. Considering this aspect and in view that our results were most distinct in patients with low Se/SELENOP concentrations, the findings may be of specific relevance to populations with insufficient Se intake. Further studies are needed to assess the results in such Se-deplete areas in comparison to Se rich countries, such as the USA, where the contribution from SELENOP-aAb to disease course may be rather marginal.

The observed prevalence of 7.65% autoimmunity to SELENOP in the patients with primary invasive breast cancer is slightly higher than reported before from patients with autoimmune thyroid disease (6.6%), and healthy subjects (0.3%), respectively. Part of this difference may be explained by the more than two-fold higher median age in this study, and the exclusive enrolment of women [39]. In how far a predisposition to breast cancer is exerted by SELENOP-aAb, or whether modified SELENOP is secreted from malignant breast tissue is unknown at present. Biosynthesis of potentially modified SELENOP by malignant cells may cause the development of SELENOP-aAb, and patients with breast cancer may consequently tend to develop autoimmunity to Se transport. Notably, the mammary gland has been described as actively secreting SELENOP, hereby enabling targeted Se supply to the offspring via mother's milk [40]. The higher prevalence observed in the patients may also be due to a higher risk of breast cancer development in the presence of SELENOP-aAb. Whether and in how far SELENOP acts as a tumour associated antigen promoting autoimmunity or whether the SELENOP-aAb rather constitute a risk factor contributing to the higher prevalence in this cohort must be investigated in further studies.

Our study has several strengths. Particularly, while most studies exploring potential clinical relevance of novel biomarkers include a rather small sample size, this investigation was conducted with one of the largest current prospective breast cancer studies in the world. As fortunately, the recurrence rate of breast cancer is relatively low, and in view that the prevalence of SELENOP-aAb is relatively moderate, the large study size was a crucial prerequisite for providing sufficient statistical power for the analyses conducted. Even though we characterize the potential prognostic relevance of very novel autoantibodies, exploration was not conducted arbitrarily, but based on prior findings that originated from the same patient group in the SCAN-B study, and without arbitrary cut-off determination. Hereby, both the congruency to our prior results on the relationship between Se and surviving breast cancer within the SCAN-B study cohort and the complete and precisely constructed database with a very low number of missing values in confounders argue for a high solidity of the findings reported. From a methodological standpoint, the large set of available covariates that were corrected for granted a focused investigation of an independent effect of serum SELENOP-aAb on mortality and recurrence. The availability of corresponding serum Se and SELENOP concentrations along with GPX3 activity levels did provide important contextual value, and was a relevant control for correct measurements of the main exposure, i.e., the SELENOP-aAb concentrations and their prognostic relevance.

Our study also has several limitations. Due to the observational study type, residual confounding cannot be fully ruled out. Even though we have controlled for the most important potential confounders of breast cancer recurrence, information on other autoimmune diseases, prior inflammatory events or other potential triggers for autoimmunity against SELENOP are missing. While in particular prevalent systemic autoimmune disease has been shown to associate with higher overall mortality, the association of autoimmunity and cancer risk and survival is not conclusive at present [41]. Thus, while it may modify the results for mortality, we do not think that it affects the main endpoint, i.e., recurrence of breast cancer. Another limitation concerns the notion that the data are retrieved from a single blood sample per patient. As the data indicate that SELENOP-aAb are associated with age, some patients who developed SELENOP-aAb within the follow-up time might have been missed. However, this limitation would rather lead to a higher association than reported, and not challenge the main results. From our experience, naturally occurring autoantibodies are relatively stable over time, once developed, supporting the notion that the initial blood sample provides relevant information for SELENOP-aAb during the time after diagnosis [42].

Beside the novel findings in relation to breast cancer, our results also outline promising paths of future research. An important aspect would be to replicate our findings in a Se rich population, such as e.g. in the USA, where the contribution from SELENOP-aAb to a poor prognosis might be minimal. The higher prevalence of SELENOP-aAb in breast cancer patients as compared to healthy subjects or Hashimoto patients implies a potential role of SELENOP-aAb in risk for developing breast cancer. This hypothesis needs to be tested in an adequate longitudinal case-control study. The dose-dependency of the results, as well as the distinctness of the findings in Se-deficient subjects indicate a causal relationship. Thus, an interventional study to test the potential benefit of Se-supplementation for correcting the deficit and poor prognosis should be considered, with applying baseline stratification for general Se deficiency and SELENOP-aAb deficiency.

## 5 Conclusion

We conclude that SELENOP-aAb are of pathophysiological relevance and provide an independent predictive value for prognosis in patients with breast cancer diagnosis. The assessment of an additional biomarker of Se status in combination with SELENOP-aAb analysis will stratify a given patient, and inform about a particularly elevated recurrence risk. The relevance of SELENOP-aAb for cancer prognosis may also apply to other malignancies, which should be tested in future analyses.

## Financial support

This study was funded by 10.13039/501100001659Deutsche Forschungsgemeinschaft (DFG), Research Unit FOR-2558 “TraceAge” (Scho849/6–2), CRC/TR 296 “Local control of TH action” (LocoTact, P17) and the BIH, 10.13039/501100017268Berlin Institute of Health, Berlin, Germany (towards the doctoral thesis of KD).

## Conflict of interest disclosure statement

LS and PS hold shares, and PS serves as CEO of selenOmed GmbH, a company involved in Se status assessment, LS is listed as inventor on a related patent application; no other relationships or activities that could appear to have influenced the submitted work.

## Data availability statement

The data generated in this study are available upon reasonable request from the corresponding author.

## Ethical approval

This study was conducted in accordance with the Declaration of Helsinki and has been approved by the Regional Ethical Review Board of Lund (diary numbers 2007/155, 2009/658, 2009/659, 2014/8), the county governmental biobank center, and the Swedish Data Inspection group (diary number 364–2010).

## Author contributions

**K. Demircan**: Conceptualization, formal analysis, data curation, software, formal analysis, investigation, visualization, methodology, writing–original draft, writing–review and editing. **Q. Sun**: Methodology, data curation, investigation, Writing-Review & Editing. **Y. Bengtsson**: Methodology, formal analysis, data curation, investigation, writing-review & editing. **P. Seemann**: Methodology, writing-review & editing. **J. Vallon-Christersson**: Data curation, resources, investigation, writing-review & editing. **M. Malmberg**: Resources, investigation, writing-review & editing. **L.H. Saal**: Data curation, resources, investigation, writing-review & editing. **L. Rydén**: Data curation, resources, investigation, writing-review & editing. **Å. Borg**: Supervision, project administration, resources, investigation, writing-review & editing. **W.B. Minich**: Methodology, writing-review & editing. **J. Manjer**: Conceptualization, supervision, project administration, resources, formal analysis, investigation, writing–original draft. **L. Schomburg**: Conceptualization, formal analysis, supervision, funding acquisition, project administration, resources, investigation, writing–original draft.
