# Supplementary material for: Autoimmunity to selenoprotein P predicts breast cancer recurrence
Source: Redox Biol. 2022 May 25;53:102346. doi: 10.1016/j.redox.2022.102346 (PMC9157254; doi:10.1016/j.redox.2022.102346)
Supplement: Multimedia component 1 [file mmc1.pdf]

Autoimmunity to selenoprotein P predicts breast cancer recurrence

Kamil Demircan[1,2], Qian Sun[1], Ylva Bengtsson[3], Petra Seemann[1,4], Johan Vallon-Christersson[5], Martin Malmberg[6], Lao H Saal[5], Lisa Rydén[3], Waldemar B. Minich[1], Åke Borg[5], Jonas Manjer[3] and Lutz Schomburg[1,*]

[1] Institute for Experimental Endocrinology, Charité-Universitätsmedizin Berlin, corporate member of Freie Universität Berlin, Humboldt-Universität zu Berlin, and Berlin Institute of Health, Berlin, Germany
[2] Berlin Institute of Health (BIH), Biomedical Innovation Academy (BIA), Berlin, Germany
[3] Department of Surgery, Skåne University Hospital Malmö, Lund University, Malmö, Sweden
[4] selenOmed GmbH, Berlin, Germany
[5] Division of Oncology, Department of Clinical Sciences Lund, Lund University, Lund, Sweden
[6] Department of Oncology, Skåne University Hospital, Lund, Sweden

*** Correspondence:** Lutz Schomburg
Institute for Experimental Endocrinology
Hessische Str. 3-4
Charité – Universitätsmedizin Berlin
10115, Berlin, Germany
Lutz.schomburg@charite.de

Supplementary Material

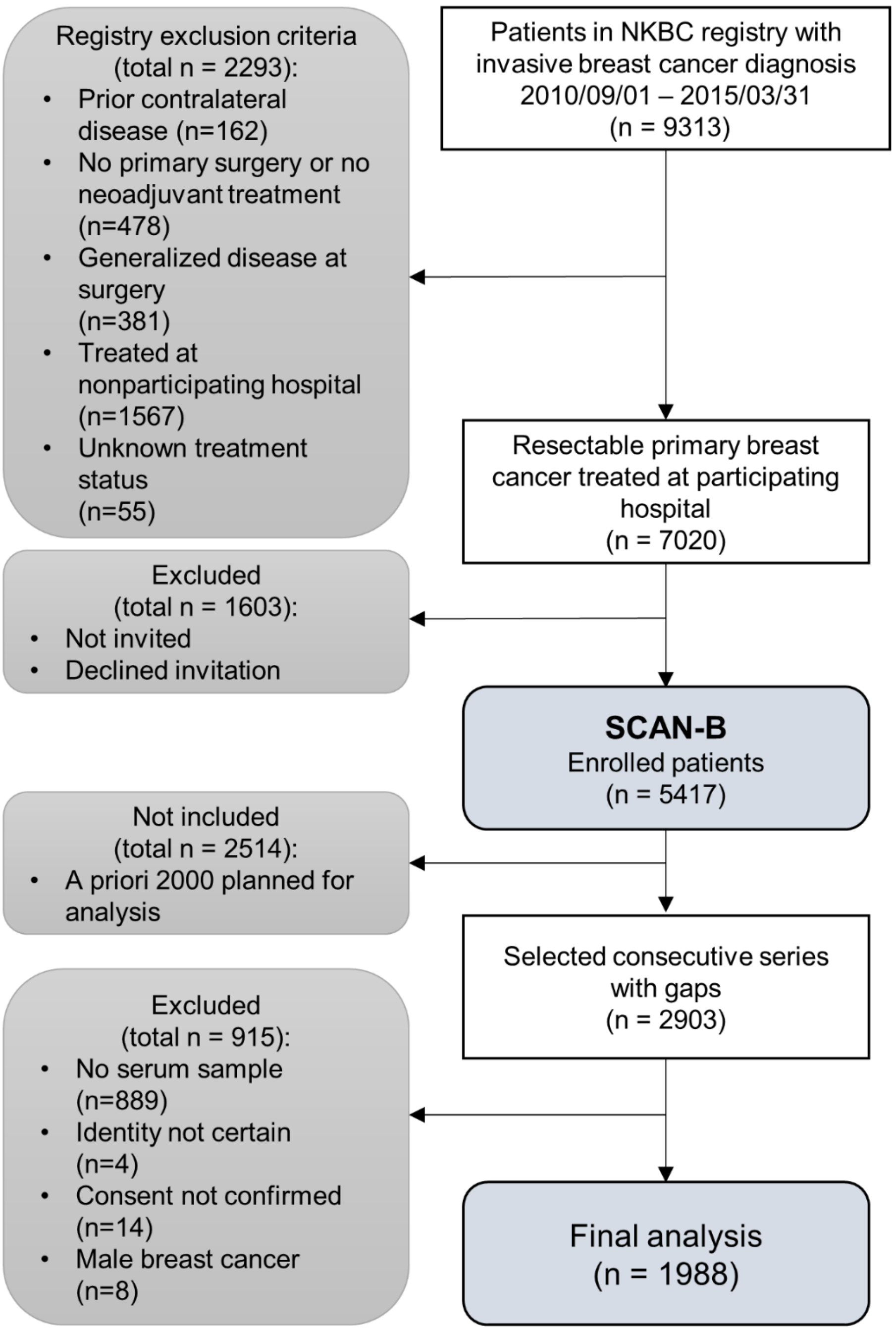

Supplementary Figure 1 Eligibility and exclusion criteria of the study population. Starting with a total set of 9313 breast cancer patients in the registry, the final number of eligible cases analysed were 1988 patients with almost complete information on survival and breast cancer recurrence.

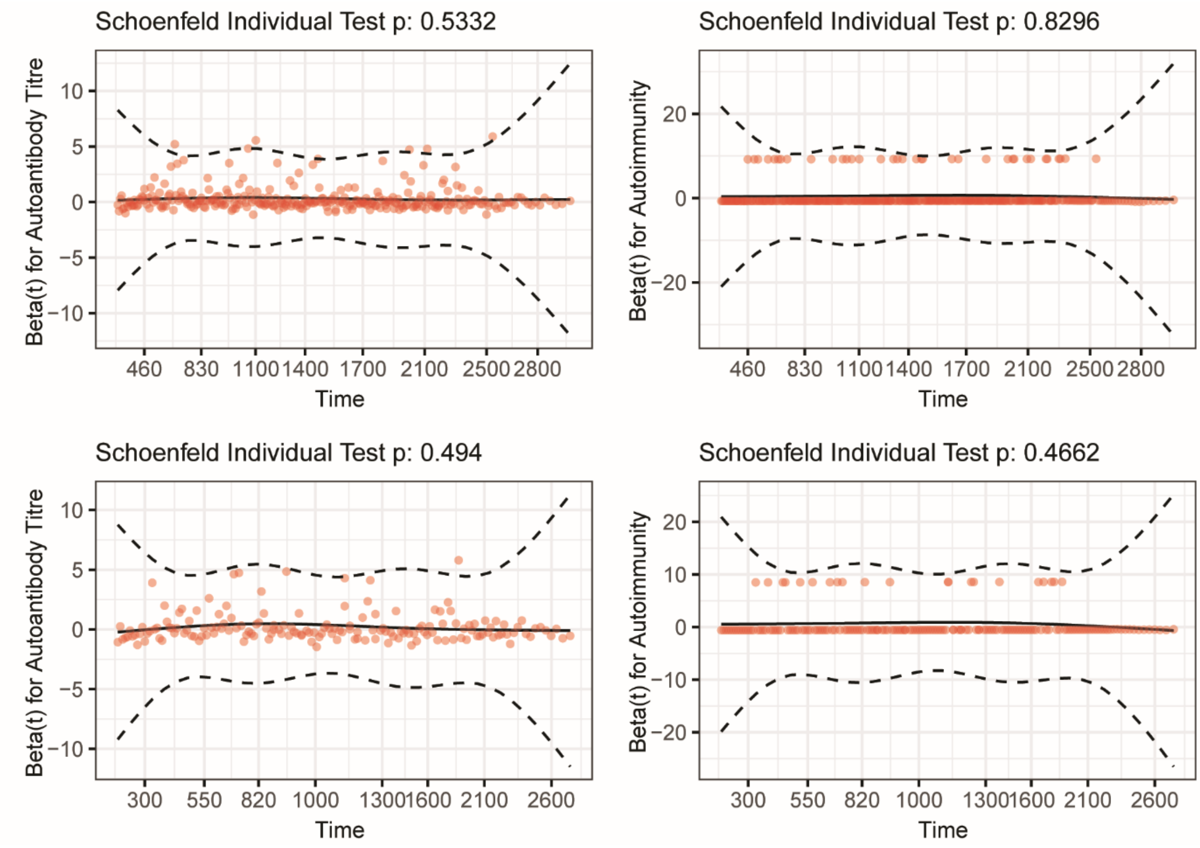

Supplementary Figure 2 Exemplary Schoenfeld residual plots for checking of proportional hazards assumption. No violations of proportional hazards assumption were observed.

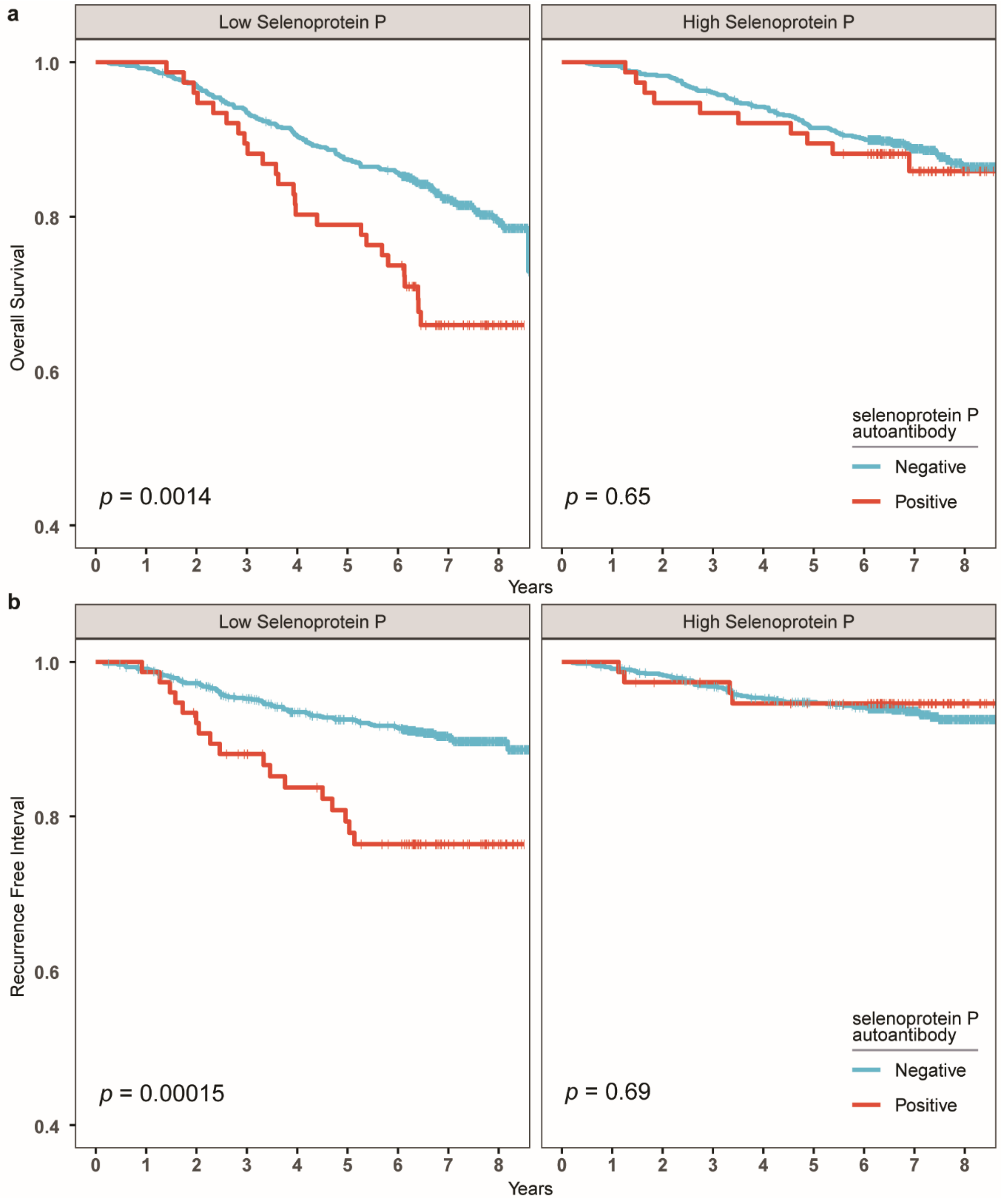

Supplementary Figure 3 Kaplan Meier plots for overall survival and recurrence free interval stratified by total serum SELENOP concentrations. a Overall survival according to autoantibody positivity was assessed with Kaplan Meier plots and log-rank test. Overall survival only differed significantly between the two groups in patients with SELENOP levels below the median of 4.08 mg/l, log-rank p=0.0014. **b** Recurrence free interval also differed only in the low SELENOP group, log-rank p=0.00015.

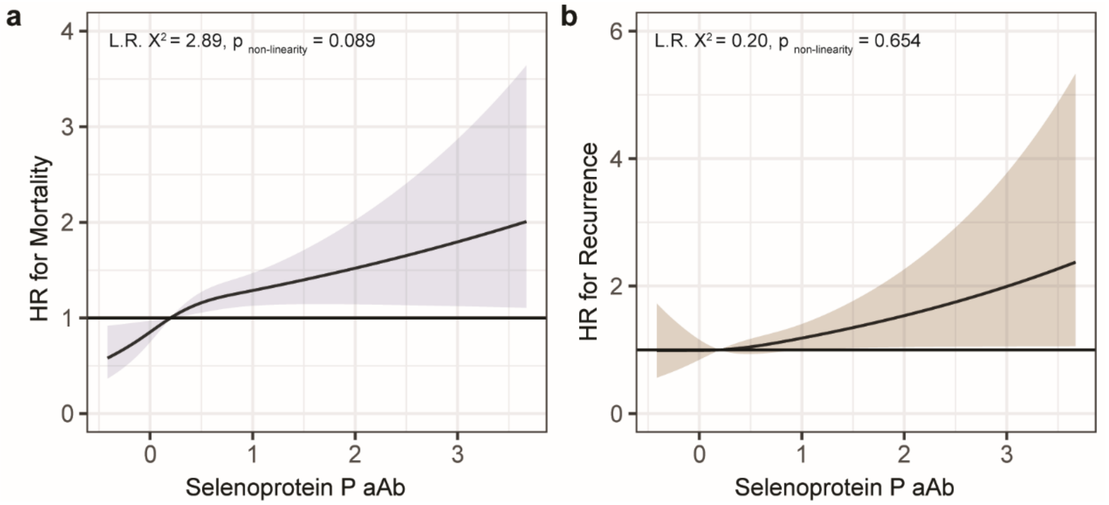

Supplementary Figure 4 Restricted cubic spline regression analysis. No deviations from linear association were observed.

Supp. Table 1 Mode of diagnosis and therapy in relation to SELENOP-aAb positivity.

Characteristic	**SELENOP-aAb negative**<br>N = 1,836	**SELENOP-aAb positive**<br>N = 152
**Diagnosis**		
Screening	970 (54%)	76 (50%)
Clinical	843 (46%)	76 (50%)
**Surgical Procedure Breast**		
Mastectomy	762 (42%)	53 (35%)
Partial Mastectomy	1,074 (58%)	99 (65%)
**Surgical Procedure Axilla**		
Sentinel Node Surgery	1,154 (63%)	111 (73%)
Sentinel Node + Clearence	418 (23%)	27 (18%)
Clearence Only	231 (13%)	13 (8.6%)
Sampling	23 (1.3%)	0 (0%)
No Axillary Surgery	8 (0.4%)	1 (0.7%)
**Endocrine Therapy**		
No	469 (26%)	49 (32%)
Yes	1,360 (74%)	103 (68%)
**Chemotherapy**		
No	1,199 (66%)	108 (71%)
Yes	630 (34%)	44 (29%)
**Immunotherapy**		
No	1,635 (89%)	138 (91%)
Yes	194 (11%)	14 (9.2%)
**Radiotherapy**		
No	623 (34%)	48 (32%)
Yes	1,206 (66%)	104 (68%)

n (%)

Missing not shown if <2%.

Supplementary Table 2 Recurrence free interval according to autoantibodies stratified by SELENOP concentrations

		At Risk	Recurrence	**Univariate** [a]		**Age Adjusted** [b]		**Fully Adjusted** [c]	
Group	SELENOP-aAb	N	N	HR	95% CI	HR	95% CI	HR	95% CI
**Low SELENOP**									
	Negative	918	86	—	—	—	—	—	—
	Positive	76	17	2.72	1.60, 4.60	2.61	1.54, 4.43	2.69	1.56, 4.64
**High SELENOP**									
	Negative	918	60	—	—	—	—	—	—
	Positive	76	4	0.83	0.29, 2.32	0.83	0.29, 2.32	0.88	0.31, 2.52

HR = Hazard Ratio, CI = Confidence Interval

a Crude model. Complete case.

b Adjusted for age at diagnosis. Complete Case.

c Fully Adjusted Model. Missing covariates were imputed using multiple imputation by chained equations. Adjusted for age at diagnosis, menopausal Status, ER expression, PGR expression, HER2 expression, Nottingham Histologic Grade, histological type, number of lymph nodes involved, modality of diagnosis, and size of tumor [mm].

Supplementary Table 3 Overall survival according to positivity of autoantibodies stratified by SELENOP concentrations

		At Risk	Death	**Univariate** [a]		**Age Adjusted** [b]		**Fully Adjusted** [c]	
Group	SELENOP-aAb	N	N	HR	95% CI	HR	95% CI	HR	95% CI
**Low SELENOP**									
	Negative	918	166	—	—	—	—	—	—
	Positive	76	25	2.02	1.32, 3.08	1.68	1.10, 2.58	1.49	0.96, 2.33
**High SELENOP**									
	Negative	918	106	—	—	—	—	—	—
	Positive	76	10	1.18	0.61, 2.27	1.16	0.60, 2.24	1.24	0.63, 2.42

HR = Hazard Ratio, CI = Confidence Interval

a Crude model. Complete case.

b Adjusted for age at diagnosis. Complete Case.

c Fully Adjusted Model. Missing covariates were imputed using multiple imputation by chained equations. Adjusted for age at diagnosis, menopausal Status, ER expression, PGR expression, HER2 expression, Nottingham Histologic Grade, histological type, number of lymph nodes involved, modality of diagnosis, and size of tumor [mm].

Supp. Table 4 Cox regression with SELENOP autoantibodies as continuous variable.

		**Univariate** [a]		**Age Adjusted** [b]		**Fully Adjusted** [c]		p non linearity [d]
Endpoint	Autoantibody continuous	HR	95% CI	HR	95% CI	HR	95% CI	
**Mortality**	per log increase	1.33	1.17, 1.52	1.27	1.10, 1.46	1.31	1.13, 1.51	0.089
**Recurrence**	per log increase	1.20	0.98, 1.47	1.19	0.97, 1.46	1.25	1.01, 1.55	0.654

HR = Hazard Ratio, CI = Confidence Interval

a Crude model. Complete case.

b Adjusted for age at diagnosis. Complete Case.

c Fully Adjusted Model. Missing covariates were imputed using multiple imputation by chained equations. Adjusted for age at diagnosis, menopausal Status, ER expression, PGR expression, HER2 expression, Nottingham Histologic Grade, histological type, number of lymph nodes involved, modality of diagnosis, and size of tumor [mm].

d Non-linear association assessed by comparing the linear fully adjusted Cox regression models to respective restricted cubic spline models (3 knots at 0.1,0.5,0.9) applying the Likelihood-Ratio test. $p_{\text{non-linearity}} < 0.05$ would indicate a non-linear relationship.

Supp. Table 5 Cox regression with SELENOP autoantibodies as log-transformed continuous variable adjusted for Se status biomarkers.

	**Fully Adjusted** [a]		**+ SELENOP**		**+ SELENOP,Se**		**+ SELENOP, Se, GPX3**	
Autoantibody continuous	HR	95% CI	HR	95% CI	HR	95% CI	HR	95% CI
per log increase	1.31	1.13, 1.51	1.28	1.11, 1.48	1.29	1.11, 1.49	1.29	1.11, 1.49
per log increase	1.25	1.01, 1.55	1.25	1.01, 1.54	1.25	1.01, 1.54	1.25	1.01, 1.54

HR = Hazard Ratio, CI = Confidence Interval

a Fully Adjusted Model. Missing covariates were imputed using multiple imputation by chained equations. Adjusted for age at diagnosis, menopausal Status, ER expression, PGR expression, HER2 expression, Nottingham Histologic Grade, histological type, number of lymph nodes involved, modality of diagnosis, and size of tumor [mm].

Supplementary Table 6 Cox regression with SELENOP autoantibodies as log-transformed continuous variable stratified by SELENOP concentrations

			**Univariate** [a]		**Age Adjusted** [b]		**Fully Adjusted** [c]	
Endpoint	Group	Autoantibody continuous	HR	95% CI	HR	95% CI	HR	95% CI
**Mortality**	Low SELENOP	per log increase	1.47	1.24, 1.73	1.36	1.15, 1.61	1.32	1.10, 1.58
	High SELENOP	per log increase	1.18	0.93, 1.49	1.15	0.89, 1.47	1.23	0.95, 1.59
**Recurrence**	Low SELENOP	per log increase	1.37	1.08, 1.74	1.34	1.05, 1.71	1.37	1.06, 1.77
	High SELENOP	per log increase	0.96	0.64, 1.44	0.96	0.64, 1.44	1.02	0.67, 1.55

HR = Hazard Ratio, CI = Confidence Interval

a Crude model. Complete case.

b Adjusted for age at diagnosis. Complete Case.

c Fully Adjusted Model. Missing covariates were imputed using multiple imputation by chained equations. Adjusted for age at diagnosis, menopausal Status, ER expression, PGR expression, HER2 expression, Nottingham Histologic Grade, histological type, number of lymph nodes involved, modality of diagnosis, and size of tumor [mm].

Supp. Table 7 Cox regression with SELENOP autoantibodies as log-transformed continuous variable stratified by selenium concentrations.

			**Univariate** [a]		**Age Adjusted** [b]		**Fully Adjusted** [c]	
Endpoint	Group	Autoantibody continuous	HR	95% CI	HR	95% CI	HR	95% CI
**Mortality**	Low Se	per log increase	1.43	1.22, 1.67	1.33	1.13, 1.56	1.32	1.12, 1.57
	High Se	per log increase	1.07	0.79, 1.45	1.06	0.78, 1.44	1.11	0.80, 1.55
**Recurrence**	Low Se	per log increase	1.28	0.99, 1.65	1.26	0.97, 1.63	1.30	0.99, 1.70
	High Se	per log increase	1.07	0.76, 1.52	1.07	0.75, 1.52	1.10	0.76, 1.60

HR = Hazard Ratio, CI = Confidence Interval

a Crude model. Complete case.

b Adjusted for age at diagnosis. Complete Case.

c Fully Adjusted Model. Missing covariates were imputed using multiple imputation by chained equations. Adjusted for age at diagnosis, menopausal Status, ER expression, PGR expression, HER2 expression, Nottingham Histologic Grade, histological type, number of lymph nodes involved, modality of diagnosis, and size of tumor [mm].

Supp. Table 8 Cox regression with fully adjusted models excluding patients with an event or censoring in the first 12 months.

Endpoint	SELENOP-aAb	HR [a]	95% CI
**Mortality**			
	Negative	—	—
	Positive	1.46	1.02, 2.11
**Recurrence**			
	Negative	—	—
	Positive	2.00	1.23, 3.25
**Mortality**	continuous per log increase	1.34	1.16, 1.55
**Recurrence**	continuous per log increase	1.29	1.04, 1.60

HR = Hazard Ratio, CI = Confidence Interval

a Fully Adjusted Model. Missing covariates were imputed using multiple imputation by chained equations. Adjusted for age at diagnosis, menopausal Status, ER expression, PGR expression, HER2 expression, Nottingham Histologic Grade, histological type, number of lymph nodes involved, modality of diagnosis, and size of tumor [mm].

Supp. Table 9 Cox regression with fully adjusted models, adjusting for treatment one by one and all treatment variables together.

	+ Breast Surgery Type [a]		+ Axillary Surgery Type [a]		+ Endocrine Treatment [a]		+ Cytotoxic Treatment [a]		+ Immuno Treatment [a]		+ Radio Treatment [a]		All Treatment [a]	
Autoantibody continuous	HR	95% CI	HR	95% CI	HR	95% CI	HR	95% CI	HR	95% CI	HR	95% CI	HR	95% CI
**Mortality** per log increase	1.32	1.14, 1.52	1.31	1.13, 1.51	1.31	1.13, 1.51	1.32	1.14, 1.52	1.30	1.12, 1.50	1.32	1.14, 1.52	1.31	1.13, 1.51
**Recurrence** per log increase	1.27	1.03, 1.57	1.26	1.02, 1.56	1.26	1.02, 1.56	1.26	1.02, 1.55	1.22	0.99, 1.52	1.26	1.02, 1.56	1.25	1.00, 1.55

HR = Hazard Ratio, CI = Confidence Interval

a Fully Adjusted Model. Missing covariates were imputed using multiple imputation by chained equations. Adjusted for age at diagnosis, menopausal Status, ER expression, PGR expression, HER2 expression, Nottingham Histologic Grade, histological type, number of lymph nodes involved, modality of diagnosis, and size of tumor [mm]. Each treatment was added individually.

Software and packages

All statistical analyses were conducted using the R language (version 4.1.2.) on the RStudio environment. Following packages were applied; *dplyr (1)* and *tidyr (2)* were used to manipulate data and data structures. *mice (3)* was used to conduct multiple imputation by chained equations. *ggplot2 (4)* and *ggpubr (5)* were used to visualize figures, and apply Wilcoxon-Rank-sum test, as well as Spearman's rank correlation test. *Survminer (6)* was used to visualize Kaplan-Meier plots, and compute p-values from log-rank test. *Survival (7)* was used to compute schoenfeld residuals. *Gtsummary (8)* was used to generate summary tables, and compute Wilcoxon-rank-sum test, Pearson's Chi-squared test and Fisher's exact test. *Rms (9)* was used to visualize restricted cubic spline regressions, and to apply likelihood ratio test.

1. Wickham H, Francois R, Henry L, Müller K. dplyr: A grammar of data manipulation. R package version 04. 2015;3:p156.
2. Wickham H, Henry L. tidyr: Easily Tidy Data with 'spread ()'and 'gather ()'Functions. R package version 08. 2018;2.
3. van Buuren S, Groothuis-Oudshoorn K. mice: Multivariate Imputation by Chained Equations in R. Journal of Statistical Software. 2011;45(3):1 - 67.
4. Wickham H. ggplot2: elegant graphics for data analysis: springer; 2016.
5. Kassambara A. ggpubr:"ggplot2" based publication ready plots. R package version 04 0. 2020;438.
6. Kassambara A, Kosinski M, Biecek P, Fabian S. Package 'survminer'. Drawing Survival Curves using 'ggplot2'(R package version 03 1). 2017.
7. Therneau TM, Lumley T. Package 'survival'. R Top Doc. 2015;128(10):28-33.
8. Sjoberg DD, Whiting K, Curry M, Lavery JA, Larmarange J. Reproducible Summary Tables with the gtsummary Package. R Journal. 2021;13(1).
9. Harrell FE. Regression modeling strategies: with applications to linear models, logistic and ordinal regression, and survival analysis: Springer; 2015.
